# 

**Published:** 1966-04

**Authors:** 


					Hi
April I960
INCORPORATING the medical journal of the south west
Vol 81 (ii) NO 300

				

